# Fractured Paths, Unrealised Potentials: Teacher Education and Equality of Challenge

**DOI:** 10.3390/bs16081334

**Published:** 2026-08-03

**Authors:** Carrie Winstanley

**Affiliations:** School of Education, University of Roehampton, London SW15 5PJ, UK; c.winstanley@roehampton.ac.uk

**Keywords:** Gifted Education, Initial Teacher Education, Equality of Challenge, high ability, interdisciplinary integration, educational neuroscience

## Abstract

Gifted Education remains a contested and unevenly integrated domain within Initial Teacher Education (ITE). Its position is further complicated by conceptual instability surrounding identification, classification, and educational equity. Despite substantial contributions across psychology, neuroscience, and education, its translation into practice is constrained by conceptual instability, fragmented disciplinary accounts, and institutional pressures toward standardisation. This paper argues that the marginalisation of diverse Highly Able learners is not primarily due to insufficient knowledge, but to a structural failure to integrate existing knowledge into a coherent account of challenge. Drawing on policy analysis and interdisciplinary synthesis, the paper shows how psychological accounts of motivation and engagement, neuroscientific evidence on experience-dependent plasticity, and educational frameworks prioritising scalable pedagogy operate in parallel rather than in synthesis. Within ITE, this fragmentation contributes to a diminished understanding of high-level challenges; currently the challenge is often reduced to a simple notion of ‘stretch’ embedded within generic differentiation or adaptive teaching. To address this, the paper elaborates on Equality of Challenge as a normative and integrative principle, with a focus on low-threshold high-ceiling approaches. It reframes challenge as a matter of distributive justice and educational entitlement, positioning appropriately demanding educational experiences as a core entitlement for all learners rather than a targeted provision. It connects disciplinary insights into a shared account of how challenge should be designed and enacted within ITE and classroom practice.

## 1. Introduction

This paper responds to the call for work examining the intersection of psychology, neuroscience, and education in support of ‘gifted’ individuals. It focuses on Initial Teacher Education (ITE) as a critical yet underdeveloped area where the needs of ‘highly able’ learners are routinely overlooked or conceptually mishandled. Situated in England, UK, ITE is a particularly significant site for examining these issues because it shapes how future teachers understand learner difference, inclusion, challenge, and educational responsibility. Although highly able learners are frequently discussed within specialist studies, their needs often remain peripheral within broader ITE frameworks and policy discussions.

This paper is intentionally conceptual and normative in orientation. It does not seek to provide a systematic review of the literature, nor to offer an empirically tested model of practice. Rather, it draws selectively on policy, educational, psychological and neuroscientific scholarship to examine how the understanding of the needs of highly able learners is positioned within ITE and to consider how questions of challenge, equity and professional responsibility are currently understood within the English context. In the paper, Equality of Challenge (Winstanley, 2004) is explored as one possible framework for rethinking challenge, equity and professional responsibility within the English context. In England, teacher preparation and early professional development are shaped by policy frameworks produced by the Department for Education (DfE), the government department responsible for education policy, teacher education, and school standards. The Core Content Framework (DfE, 2019a, updated 2024) and Early Career Framework (DfE, 2019b, updated 2024) provide nationally prescribed expectations for teacher development during initial preparation and the early years of professional practice.

The central argument of this paper is that the marginalisation of highly able learners within ITE is not primarily the result of a lack of knowledge about learning and development. It reflects, instead, the fragmentation of relevant insights across psychology, neuroscience, educational theory and policy. Allied concerns have been raised within educational neuroscience, where challenges of interdisciplinary translation and integration continue to be identified despite growing bodies of research (Thomas et al., 2019). Consequently, understandings of challenge often remain conceptually underdeveloped within ITE. The paper therefore explores Equality of Challenge as a normative framework through which challenge, equity and professional responsibility might be reconsidered within ITE, reframing challenge as educational entitlement for all, rather than enrichment for a select few.

ITE can be reductively misunderstood as a neutral training environment and experience. However, it is a formative and determinative process in which understandings of cognitive diversity are actively shaped, stabilised, or marginalised. Within this space, giftedness is not absent. Instead, it is constructed through prevailing assumptions about the nature of learning and learners, recommendations concerning the efficacy of different pedagogical approaches, understandings and expectations around inclusion, un/acceptable views on equity, and notions of professional responsibility (Ofsted, 2019). By drawing together insights from psychology, neuroscience and education the paper seeks to reconsider how Gifted Education (GE) is conceptualised within ITE and how diverse highly able learners might be better understood and supported.

### Definitions, Scope and Core Concepts

As with most research papers, this work reflects the context in which it has been developed, which in this case is England within the wider UK context. (Although the paper uses the term ‘ITE’ throughout, the discussion extends into the Early Career Teacher period where relevant, recognising teacher formation as a process that continues beyond qualification.) Whilst some of the issues discussed may resonate beyond England, the analysis and claims advanced here are primarily situated within English policy and Initial Teacher Education. Across contemporary ITE in England, cognitive difference is routinely acknowledged but often only in broad and indefinite ways. Learner variability is typically addressed through general frameworks of differentiation and adaptive teaching, such as Standard 5 in the Teachers’ Standards where teachers are required to ‘adapt teaching to respond to the strengths and needs of all pupils’ (DfE, 2011, updated 2021, p. 11).

Additionally, it is relatively common for Teacher Educators, school leaders and teachers to respond to the idea of giftedness negatively. For example, those advocating for disadvantaged potential high achievers can be met with scepticism or dismissal, since the field of giftedness is often wrongly characterised as elitist and thereby threatening to equitable inclusion and their needs ‘may not be well known or, sometimes, even acknowledged’ (Melander, 2025). Ideas underpinning the notion of GE are rarely interrogated in ITE and there are indications that affective, ideological and political responses to giftedness may influence how the topic is positioned within ITE. Since training experiences are formative in shaping how (or whether) teachers understand giftedness, this needs to be addressed.

In this paper, giftedness is not treated as a fixed category, diagnostic label, or privileged status. Instead, this paper is positioned within a psychological, neuroscientific, and educational convergence that reframes HA in terms of need for challenge, rather than fixed, intrinsic ‘giftedness’. Rather than giftedness as a stable category, the focus is on differential ease of acquisition, where, at specific points in their development, learners differ substantially in the ease and speed with which they acquire particular forms of knowledge and skill, and the levels of support they need, across all the different domains typically found in schooling, whether, for instance, the learning is physical, linguistic, mathematical, geographical, etc. This includes learners from diverse social, cultural and linguistic backgrounds, as well as those with uneven developmental profiles, including dual and multiple exceptionalities (DME), where high ability coexists with Special Educational Needs/Disability (SEN/D), neurodivergence, or other forms of learning difference.

From this perspective, lack of appropriate challenge is more than pedagogical inefficiency; it can influence learners’ achievements and also shape long-term patterns of engagement, including motivation, self-concept and identity formation. This is unsurprising given ‘students and teachers engage social, emotional and cognitive processes all at once’ (Fischer & Bidell, 2006, as cited in Immordino-Yang et al., 2019), meaning that experiences of challenge are simultaneously cognitive, social and emotional. Contemporary interdisciplinary perspectives similarly highlight the importance of educational opportunity and environmental conditions. Indeed, ‘in sub-optimal environments, measures of environmental quality and learning opportunities overwhelmingly swamp the predictive power of genes’ (Bates et al., 2013, as cited in Immordino-Yang et al., 2019, p. 9). Insufficient challenge also raises questions of educational justice because it reflects how education systems define and respond to variation in the speed, depth, and pattern of learners’ engagement with knowledge, skills and understanding. The analysis is further informed by Biesta’s (2010) work on the purposes of education, which provides an important lens through which to examine the relationship between evidence, educational judgement and professional responsibility.

Equality of Challenge (Winstanley, 2004) is used here as a normative and conceptual framework through which to consider whether learners are entitled to appropriately calibrated challenge, shaped by their developmental level, their attainment in particular areas of learning, and their motivational orientation. It is explored as one possible response to fragmented accounts of learner difference and as a way of addressing the ethical and political tensions that arise when provision is framed through narrowly stratified or potentially elitist interpretations of ability. Challenge is a condition of meaningful engagement with learning itself, rather than being positioned as a reward for prior attainment. This reframes the central question inclusively, shifting from asking who is identified as gifted to considering what counts as adequate intellectual demand across the full distribution of learners.

Equality of Challenge (EoC) offers one possible way of integrating psychological accounts of motivation, connecting identity, and developmental need; neuroscientific perspectives relating to plasticity, under-stimulation, and cognitive demand; and educational concerns with curriculum, pedagogy, and assessment. It does so while drawing on Biesta’s (2010) distinction between three functions of education: qualification (the transmission and acquisition of knowledge, skills, and dispositions), socialisation (becoming part of existing social, cultural, and institutional orders), and subjectification (the process of becoming a subject who acts and takes responsibility rather than simply fitting into established norms). In this sense, the argument reframes giftedness not as a discrete specialist domain within education systems, but as a lens through which to examine the ethical and epistemic assumptions that structure teacher education itself.

Note that this paper does not examine in detail practice issues such as the distinction between differentiation and adaptive teaching in the classroom. While important for pedagogical decision-making, these constructs are treated here only insofar as they relate to how cognitive demand is framed and operationalised within teacher education, rather than as practice-level models in their own right. It also does not engage directly with dual or multiple exceptionalities (DME), despite their obvious relevance to cognitive variability and uneven developmental profiles. This is a deliberate boundary, reflecting the paper’s focus on structural and conceptual conditions shaping the specification of challenge within ITE, rather than the classification or instructional support of specific learner groups.

The paper first examines how highly able learners are positioned within contemporary Initial Teacher Education and considers why they often remain marginal within broader discussions of inclusion and learner diversity. It then explores the conceptual and political tensions surrounding Gifted Education, including debates concerning classification, identification and equity. The paper subsequently considers how psychology, neuroscience and education frequently operate as parallel rather than integrated conversations around challenge and learner development. Finally, Equality of Challenge is explored as a normative framework through which challenge, educational justice and professional responsibility might be reconsidered within teacher education.

## 2. The Marginalisation of Highly Able Learners in Initial Teacher Education

### 2.1. Highly Able Learners and the Formation of Teachers

The marginalisation of Gifted Education within ITE is reinforced through policy and institutional systems that prioritise what is measurable, standardised, and inspectable (Montacute, 2018). Within such systems, visibility is shaped by requirements for accountability, inspection, and system-level comparison and is not neutral (Ball, 2003; Biesta, 2010). This affects how cognitive difference is framed, particularly at the upper end of attainment, where learning demands are less easily captured through standardised measures. Across policy and research, high-attaining and highly able learners are not absent by accident, but given comparatively less visibility within ITE frameworks revealing the persistent assumption that HA is synonymous with low need. Institutional design limits the prominence of sustained cognitive challenge at the upper end of attainment. Inspection frameworks shape what becomes visible within ITE and schools, and current ITE inspection criteria do not routinely require explicit consideration of highly able learners as a distinct group (Ofsted, 2019).

Inspection criteria used in the Ofsted regulation of ITE prioritise curriculum coherence, subject knowledge, and adaptive teaching, but do not require explicit or sustained attention to high-ability learners (Ofsted, 2019). While these frameworks address learner variability through general principles of inclusion and responsiveness, they do not explicitly specify HA or GE as a discrete or distinct areas of focus. Consequently, high-level cognitive demand remains largely implicit within inspection criteria, with what is assessed tending to shape what is prioritised in practice. Indeed, schools are asked to ‘stretch’ students, a less specific formulation than an earlier Ofsted requirement (Ofsted, 2015), which explicitly demanded a focus on ‘whether more able pupils in general and the most able pupils in particular are achieving as well as they should’ (Loft & Danechi, 2020, p. 13). ‘The 2015 Ofsted document has been formally withdrawn, yet it had influenced policy in schools and so merits noting here, in this discussion around the difficulty of supporting pupils with contested definitions. So, forms of challenge associated with advanced learners are typically embedded within broader pedagogical descriptors rather than specified as a distinct focus, reinforcing the argument that educational priorities may become (reductively) shaped by what is measured and inspected.

The Core Content Framework (CCF) and Early Career Framework (ECF) set out the core entitlement for teacher development in England, spanning initial preparation and the early years of professional practice. Teacher identity within the CCF and ECF is shaped through an emphasis on becoming a professionally accountable practitioner who delivers and refines curriculum within a structured pedagogical system (DfE, 2019b, 2024). Across initial training and early career development, this positions teachers primarily as implementers of broadly applicable strategies relating to behaviour management, assessment, curriculum sequencing, and adaptive teaching, rather than as designers of differentiated intellectual experiences. This broader tendency is also reflected in recent educational neuroscience scholarship, which notes that ‘most ITT programmes lack a comprehensive focus on the neurocognitive mechanisms of learning’ (Thomas & Arslan, 2025, p. 310). This also has implications for how teachers understand learner variability, particularly whether they see themselves as primarily needing to adjust learning in response to their learners, or to enact and deliver a pre-determined curriculum through generalised responsiveness.

Within this professional identity structure, learner difference is addressed through inclusive but generalised accounts of adaptation, in which HA is incorporated into a single continuum of differentiation (DfE, 2024). This may flatten distinctions in learner variation, potentially shaping classroom practice through sidelining qualitatively distinct forms of intellectual engagement. This may be particularly significant for learners with DME, whose high attainment potential and additional learning needs can both become obscured within broad categories of provision. This orientation is consistent with the Teachers’ Standards, which define professional responsibility in terms of meeting the needs of ‘all pupils’ (DfE, 2021, p. 11). While this language affirms inclusion, it reinforces a universal model of practice in which differentiation is embedded within general teaching competence rather than developed as a specialist capacity. National guidance on disadvantage similarly extends this framing, stating that Pupil Premium funding should support all learners, ‘including nurturing the more able pupils to excel’ (DfE, 2015, p. 3), further consolidating an integrated model of provision in which HA is situated within mainstream professional responsibility rather than treated as a distinct pedagogical domain.

This professional framing shapes how theoretical knowledge is engaged with during teacher education and early career development. Orchard et al. (2020) describe teacher formation as involving both ‘head and heart’ work, where conceptual understanding and relational practice are intended to develop together (p. 142). However, in ITE and Early Career Development, engagement with the ‘academic dimension’ is constrained by limited time, workload pressures, and the ‘busyness’ of school life (Orchard et al., 2020, pp. 145–147), with theory often becoming secondary to immediate classroom demands. Thomas echoes this picture, observing that teachers often encounter new pedagogical approaches within a context dominated by ‘workloads, funding cuts to school budgets, and student behaviour in the classroom’ (p. 253). Similarly, Arslan and Thomas observe that ‘the crowded ITT curriculum often limits the depth of coverage and details of application’ (p. 311), even where such relevant knowledge is formally included. These observations reinforce Orchard et al.’s (2020) argument that, although dialogic and accessible approaches can support engagement, this remains unevenly embedded in everyday professional conditions (p. 148). These conditions align with the assumptions embedded in the CCF (DfE, 2019a) and ECF (DfE, 2024). Together, these frameworks and the conditions of practice described by Orchard et al. (2020) suggest that teacher formation occurs within environments shaped by workload, accountability, and immediacy, conditions that may constrain sustained engagement with complex questions of learner difference and challenge.

### 2.2. Evidence and High Attainment

Evidence from large-scale datasets and sector-level studies and guidance suggests that the problem is substantive. Persistent gaps exist between the needs of HA learners and the extent to which ITE frameworks are managing to develop the knowledge and confidence required to meet those needs. The Education Endowment Foundation (EEF) ‘Teaching and Learning Toolkit’ (2021) reflects an emphasis on scalable, evidence-informed strategies such as feedback, metacognition, and retrieval practice. The Education Endowment Foundation’s Teaching and Learning Toolkit is a widely used evidence resource that synthesises findings from large numbers of educational studies and meta-analyses, providing schools and policymakers with estimates of the relative effectiveness of different pedagogical approaches. While these approaches are robust within their evidential base, they are derived primarily from population-level effects and therefore favour interventions that improve outcomes for most learners. As a result, they are less explicitly oriented towards the upper end of attainment as a distinct pedagogical concern, with greater emphasis on strategies that produce reliable gains across the majority of pupils than on sustaining appropriately high levels of cognitive demand across the full attainment range. This general orientation is also reinforced by EEF guidance on metacognition and self-regulated learning, which frames implementation in broad classroom terms rather than differentiated accounts of attainment level (EEF, 2018).

Professional evidence points to similar patterns in teacher preparation. Loft and Danechi (2020) report persistent inconsistency in provision for HA pupils, alongside uneven coverage of this area within ITE and CPD. The National Association for Able Children in Education (NACE), a UK professional body and advocacy organisation focused on provision for more able learners, similarly highlights variability in how GE is understood and embedded across schools and training contexts, including in its ‘Breaking Barriers’ guidance (Winstanley, 2019). NACE works with schools and educators to improve provision for more able learners, making it an influential voice in debates concerning challenge, attainment, and educational opportunity (https://www.nace.co.uk/, accessed on 1 July 2025). NACE therefore offers not just evidence of variation in provision, but also insight into an uneven conceptualisation within teacher education and professional guidance. This indicates inconsistency across ITE and other professional contexts, and reveals a lack of sustained conceptual focus within both teacher preparation and policy guidance. Outcome data suggest that these conditions have practical consequences. The Sutton Trust identifies persistent underachievement among HA pupils, particularly those from disadvantaged backgrounds (Montacute, 2018). This suggests that HA learners do not consistently experience the conditions required to convert potential into attainment and to consolidate attainment into continued development within current systems, especially where access to sustained challenge is uneven. International evidence from OECD TALIS similarly highlights variability in teachers’ preparedness to provide appropriate challenge (OECD, 2019). OECD TALIS data further suggest that teachers differ considerably in their preparedness to respond to learner variability, highlighting the importance of professional knowledge and training. This reinforces the argument that provision for highly able learners depends not only on individual teacher practice, but also on how challenge is conceptualised and prioritised within teacher education systems. This suggests that capacity to respond to the upper end of cognitive variability differs across contexts, reflecting variation in professional preparation and system-level support. Taken together, these findings indicate that support for high attainment is inconsistently developed across evidence use, teacher preparation, and classroom practice.

It can be seen that research, policy and practice-based scholarship reinforce this picture by linking learner outcomes to underlying assumptions about ability, expectation, and educational opportunity. This argument is further supported by developmental and longitudinal perspectives, which suggest that the consequences of insufficient challenge may extend across learners’ educational trajectories (Freeman, 2010; Gross, 2004; Subotnik et al., 2011). These developmental perspectives sit alongside broader concerns about opportunity structures and professional understandings of challenge. At a system level, the Sutton Trust highlights persistent underachievement among high-attaining pupils, particularly those from disadvantaged backgrounds, indicating that high potential is not consistently transformed into meaningful educational achievement within current structures of opportunity and support (Montacute, 2018). At the level of practice, NACE highlights the considerable inconsistency in how HA learners are conceptualised across schools and ITE, and how challenge is understood and enacted (Winstanley, 2019).

Across developmental accounts, Subotnik et al. (2011) argue that giftedness is best understood through trajectories of talent development in which sustained cognitive challenge is central to long-term expertise formation, rather than as a static category of ability (Subotnik et al., 2011). In related longitudinal perspectives, the work of Gross (2004) and Freeman (2010) demonstrate how outcomes for the HA are shaped by environmental conditions, including the quality and consistency of educational support over time and the deleterious impact of insufficient challenge. These accounts suggest that the marginalisation of diverse HA learners is embedded in assumptions about ability, expectation, and educational value, shaping both opportunity structures and developmental trajectories. As a consequence, opportunities for sustained challenge remain unevenly distributed across educational settings and stages of development.

### 2.3. Conceptual Instability in Gifted Education

Gifted Education has an image problem. It occupies an uneasy position within education discourse, shaped by persistent debates surrounding identification, classification, equity and educational purpose. Dai (2018) explicitly describes the field as engaged in a ‘continual effort to define itself intellectually, socially, and practically’ (p. 3) and when Worrell et al. (2019) note that ‘the discourse on gifted students in the United States can be contradictory and fractious’ (p. 552), they are echoing what also rings true in many other countries. Characterised by conceptual and methodological tensions, it is unsurprising that the field continues to attract scepticism, making it difficult to build a coherent argument for how to support trainee and newly qualified practitioners. Instability is not just disagreement; it prevents shared operational use in ITE. The emotional climate surrounding giftedness may further compound these difficulties, shaping how the field is represented within teacher education.

Various concerns combine to explain the marginal and superficial treatment of GE within ITE. Primarily, contested definitions and unresolved debates around identification have made it difficult to position ‘giftedness’ as a coherent concept, limiting clarity over what should be addressed in professional preparation. Student teachers are typically exhorted to consider nuanced approaches to learners, including developmental variation and adaptive teaching (DfE, 2011). They are advised to use responsive pedagogies (Wiliam, 2018) and inclusive approaches that tend to reject fixed ability groupings in favour of extending learning opportunities for all (Glazzard & Stones, 2020). These approaches sit uneasily alongside persistent binaries such as gifted/non-gifted and inherited models of ability, which reflect conceptual ambiguity within the field (Ambrose et al., 2012).

Pfeiffer (2013) highlights the proliferation of competing definitions of giftedness, linking this directly to difficulties in identification. He also notes that assessment practices in GE can ‘run counter’ to established principles of diagnostic practice (2013), reinforcing conceptual uncertainty within ITE contexts. Lowe (2017) similarly demonstrates that there is no universally agreed definition of HA, with no requirement for schools to maintain consistent identification systems, resulting in widespread diversity in how cohorts are defined and identified across contexts. For ITE, this means that teacher educators are often required to prepare future teachers for learner differences that remain conceptually contested and inconsistent in practice.

### 2.4. Critiques of Classification and Identification

Despite sustained critique, identification practices in many contexts continue to rely heavily on psychometric testing, including IQ scores and attainment thresholds, reinforcing a fixed and measurable conception of ability (Borland, 2005; Sternberg, 2005). While a detailed discussion of alternative theories of intelligence lies beyond the scope of the present paper, these issues have been explored elsewhere (Winstanley, 2004). The focus here is less on resolving debates about the nature of intelligence than on examining how conceptual uncertainty shapes teacher education, identification practices, and educational provision. Critics argue that continued reliance on problematic psychometric testing entrenches categorical thinking and lends the appearance of objectivity to decisions that remain normatively contested, shaping policymakers’ and practitioners’ interpretations and understanding of GE (Borland, 2005; Sternberg, 2023). Within this critique, GE cannot develop as a field while it continues to delegate judgement about educational value to pre-existing instruments, rather than interrogating what should count as ‘giftedness’ and problematising the concept itself. A persistent zero-sum framing remains, in which additional provision for HA learners is assumed to reduce resources or attention available to others, reinforcing concerns about elitism and inequity.

Sternberg (2023) reframes giftedness as fundamentally a social labelling process rather than a discovered trait, emphasising that definitions are constructed through choice rather than revealed through measurement. From this perspective, reliance on readily available indicators such as IQ scores, attainment thresholds, or GPA is characterised as epistemically unreflective, as it delegates substantive judgement about educational value to pre-existing instruments rather than interrogating what should count as ‘giftedness’ in the first place.

Borland has long advanced a strong critique of classification itself, arguing that ‘giftedness’ is not coherent. He depicts it as a socially constructed label embedded within historically contingent schooling systems, and that identification processes actively produce categories that may reproduce inequality (2014). However, he also retains a commitment to educational responsiveness, noting that educators must address the ‘inescapable fact of individual differences in how and how well students learn at a given time in a given subject’ (Borland, 2005, p. 1). Sympathetic to Borland’s ideas, Ambrose et al. (2012) identify the unhelpful persistence of rigid binaries such as gifted/non-gifted, arguing for more dynamic, interdisciplinary understandings that could capture cognitive complexity. By titling their 2012 volume ‘Confronting Dogmatism’, (Ambrose et al., 2012), they foreground the extent to which Gifted Education is characterised by ingrained and competing orthodoxies, reinforcing on-going conceptual fragmentation within the field.

GE is characterised by persistent conceptual instability rather than definitional convergence. Dai (2018) describes the field as engaged in a ‘continual effort to define itself intellectually, socially, and practically’ (p. 3), stating that it has an ‘identity crisis’ (p. 16). He further argues that giftedness is context-dependent and so identification is inherently unstable, referring to the ‘arbitrariness of gifted identification’ (Dai, 2018, p. 16). These dynamics are shaped not only by psychological theory but also by broader social and political forces.

GE appears, then, less as a unified field and more as a site of sustained epistemological tension, where classificatory language remains unsettled. These different accounts indicate conceptual instability, definitional fragmentation, ambiguity, confusion and a lack of consensus across the field. These appear less as temporary limitations than as recurring features arising from competing epistemologies, policy pressures, and normative assumptions about ability, value and educational entitlement. The instability is structural. This helps explain why GE is often marginalised, treated superficially, or simply subsumed within broader categories, such as differentiation or adaptive teaching. It is difficult to treat a contested idea as a clear, distinct domain within a system oriented towards clarity, accountability, and standardisation. Together with the associated ethical and political tensions, the result of this conceptual slippage is that cognitive difference remains acknowledged but insufficiently operationalised within ITE, research and policy. Viewed in this way, resistance to GE is not necessarily irrational opposition to evidence, but may reflect deeper uncertainties about classification, value and educational purpose.

## 3. Fragmentation Across Disciplines and the Absence of Integration

This complexity is reinforced by fragmentation across disciplinary domains. The field is characterised less by a lack of knowledge than by a failure of synthesis across psychology, neuroscience, and education, where robust but partial accounts of high ability rarely converge in ways that meaningfully shape professional preparation. Instead, these perspectives circulate in parallel, producing conceptual richness without corresponding synthesis and integration in teacher education practice.

Psychological accounts emphasise cognition, motivation, identity, and self-regulation. HA learners are often characterized in terms of qualitative differences in engagement, motivation, self-regulation and self-concept (Zimmerman, 2000). They are not reducible to attainment measures or performance thresholds; engagement, self-concept, and responsiveness to learning environments and teachers are also important (Dai, 2018). A sustained mismatch between learner capacity and curricular demand is associated with under-stimulation and disengagement (Bjork & Bjork, 2011; Subotnik et al., 2011).

Neuroscientific perspectives highlight plasticity and experience-dependent development, suggesting that cognitive growth is shaped by appropriately calibrated environmental challenge. Thomas (2024) argues that ‘education must be construed in terms of multiple interacting levels, each equally influential in children’s educational outcomes’ (p. 254), cautioning against reductionist accounts that privilege any single explanatory framework. However, translation into pedagogical design remains limited due to an enduring gap between explanatory models and educational implementation (Howard-Jones, 2014). Thomas et al. (2024) similarly emphasise the developmental and contextual nature of learning, while Thomas and Arslan (2025) draw attention to the continuing difficulties of translating neuroscientific insights into teacher education and classroom practice.

As Howard-Jones (2014) notes, ‘in education, effective communication may require neuroscientists to work in collaboration with those who are more familiar with the cultural conditions and concepts of education—that is, the educators themselves—to ensure that the content of the communication is fit for purpose’ (p. 6). This challenge persists. Thomas et al. (2019, p. 487) note that while educational neuroscience has generated considerable interest, “translation into the classroom is also hard”, highlighting the continuing difficulty of integrating disciplinary knowledge into educational practice and teacher preparation. They further observe that ‘a broader understanding of social and political factors is required, rather than just cognitive neuroscience factors’ (Thomas et al., 2019, p. 487), reinforcing the argument that questions of challenge and educational opportunity extend beyond individual cognition alone.

Together, these domains provide complementary but disconnected accounts of cognitive variability. Thomas (2024) cautions that educational neuroscience “should not be viewed as an attempt to reduce education to the individual or to biology” (p. 251), since learning is shaped by interacting child, school, family, societal and policy factors. The challenge, therefore, is not to privilege one disciplinary perspective over another, but to bring these different accounts into a more productive relationship.

Recent educational neuroscience scholarship examines how relationships between neuroscience and educational practice might be strengthened (Thomas et al., 2024). Indeed, Thomas and Arslan (2025) argue that many attempts to introduce neuroscience into education involve a ‘one-way translation of neuroscientific knowledge to classroom practice’ (p. 311), rather than a reciprocal exchange between researchers and practitioners.

Education and policy frameworks prioritise scalability, accountability, and generalisable pedagogical strategies, privileging what can be standardised across populations rather than differentiated across cognitive profiles (Biesta, 2010; Montacute, 2018). Within such systems, adaptive teaching and intervention strategies are typically derived from population-level evidence (EEF, 2021), rather than finely grained accounts of variation, with inspection frameworks reinforcing this by shaping what is prioritised in practice (Ofsted, 2019). This contributes to a narrowed model of challenge, in which expectations are typically calibrated towards mid-range attainment rather than differentiated across the full range of cognitive variability.

Taken together, perspectives illuminate cognitive variability but do not, in themselves, provide a coherent basis for understanding and enacting appropriate challenge. Within ITE, they are rarely brought into alignment in ways that inform curriculum design, pedagogical decision-making, or the design of appropriate challenge. This produces a structural disjunction between explanation and practice: what is known about learning does not consistently inform how learning environments are constructed. For GE, this compounds the conceptual instability identified earlier. Without integration across disciplinary accounts, ITE lacks a coherent professional language for articulating cognitive difference and designing appropriate challenge. Consequently, reform is unlikely to be achieved simply through adding further content.

The issue is not only marginalisation of GE as a topic, but a structural disconnect in the knowledge base required to support it, reflecting a failure of synthesis, not an absence of evidence. Thomas et al. (2019) further argue that educational neuroscience must address the “academic inertia that produces resistance to interdisciplinary research” (p. 487), a challenge that resonates with the fragmented relationship between psychology, neuroscience and education described here. It is within this fragmented epistemic and institutional landscape characterised by conceptual slippage, that a more integrative account becomes necessary. The following section develops this through the concept of Equality of Challenge (Winstanley, 2004), drawing together insights from psychology, neuroscience, and education into a normative framework for understanding how to support the full range of learners.

### 3.1. Equality of Challenge

Equality of Challenge (EoC) is introduced as a conceptual response to the fragmented treatment of learner variability, challenge and cognitive demand across psychology, neuroscience, and education within ITE. Originally articulated by Winstanley (2004), Equality of Challenge is used here not as a new framework, but as an integrative and normative concept for thinking about challenge, learner variability and educational responsibility. It emerges from the argument that insights into cognitive and other variability exist across disciplines but remain insufficiently integrated into a coherent framework capable of informing pedagogical design and professional judgement.

EoC is not proposed as a new typology of giftedness or a discrete model of learner classification. It functions as a bridging concept, connecting psychological accounts of engagement and self-regulation, neuroscientific evidence on experience-dependent development, and educational understandings of curriculum and pedagogy and linking these different perspectives through a shared concern for providing appropriate challenge. EoC therefore reframes equality not as uniformity of provision, but as the principled distribution of challenge. It moves beyond approaches to ITE that treat ‘stretch’ as implicit or optional, arguing instead that appropriate challenge must be considered explicitly. As Thomas (2024) notes, ‘development is not a passive process that happens to a child, rather it is experience driven’ (p. 253).

This position also raises questions of educational value that cannot be resolved through psychological or neuroscientific evidence alone. Biesta (2010) argues that explanations of how learning occurs do not, by themselves, determine what education ought to value. He critiques effectiveness-driven ‘learnification’ approaches to education, arguing that educational purposes cannot be reduced to questions of learning alone. Although psychology and neuroscience provide important insights into cognition, motivation and development, they cannot specify what constitutes worthwhile challenge, meaningful engagement, or educationally significant experience. In policy and practice, these explanatory accounts are often ‘operationalised’ through ‘what works’ interventions that privilege measurable gains, average effects, and standardised improvement. EoC therefore foregrounds questions about the nature and quality of the challenge encountered by learners, instead of focusing solely on attainment or rate of progress.

If the central issue is not absence of knowledge but failure of integration, then reform requires a reconfiguration of how psychological, neuroscientific, and educational insights are operationalised within teacher education. Rather than expanding content, the focus shifts to how cognitive demand is understood, specified, and enacted across learners. Building on the interdisciplinary perspectives outlined above, appropriately calibrated challenge can be understood through four interrelated principles. First, psychological accounts position cognitive variability as central to learning, suggesting that differences in engagement, motivation, and self-regulation must inform how progression is understood within classrooms. Second, neuroscientific evidence on plasticity implies that cognitive development depends on appropriately calibrated challenge, where sustained under-stimulation risks limiting higher-order development. Third, educational practice requires clearer specification of cognitive demand within curriculum and pedagogy, as generalised notions of ‘stretch’ do not reliably translate into consistent pedagogical expectations. Fourth, ITE requires a shared conceptual language through which cognitive demand can be identified and enacted across disciplines and stages of training. Without this, understanding remains fragmented and inconsistently applied in professional judgement.

At the core of EoC is a distributive conception of educational justice that redefines equality not as uniform provision, but as the equitable structuring of challenge across the full range of learners. As I have argued elsewhere, all pupils merit Equality of Challenge (Winstanley, 2004, 2008, 2010). Merry (2008) similarly states: ‘the gifted are owed what all children are owed, namely, a quality education that adequately challenges them. […]: to be adequately challenged is to be presented with tasks that demand substantial growth in ability, understanding and the ability to flourish (p. 57). EoC redefines challenge as a matter of distributive cognitive justice rather than procedural equality. Equality is not achieved through sameness of provision, but through the equitable distribution of cognitive demand across learners. The expectation that all learners experience sufficient challenge to remain cognitively engaged reframes challenge as an entitlement embedded within all pedagogy, rather than an optional extension for selected learners. EoC therefore shifts the focus from procedural delivery to the just distribution of learning opportunity across the full attainment range.

It is also important to note that EoC is not presented here as a formally validated programme or intervention. Rather, it represents a conceptual approach that has evolved through sustained engagement with educational practice since its original articulation (Winstanley, 2004). Developed through professional dialogue and application across a range of educational contexts, including inclusive settings, it is offered as a normative and heuristic concept for thinking about challenge, rather than as a prescriptive model with fixed procedures.

### 3.2. EoC in Practice

This section presents the ingredients or principles for EoC in practice. Having established EoC as a normative framework, this section considers the mechanisms through which appropriately calibrated challenge may influence learning and development in educational practice. The model has further elements, but most relevant here are:

− cognitive engagement and cognitive dissonance (including Low-Threshold High-Ceiling tasks)
− risk of failure and chance of success
− independence and self-direction
− metacognition

Together (alongside additional principles such as peer relationships, novelty and learning beyond the classroom,) these principles explain how learning is shaped by the alignment between task demand and current learner capability (Winstanley, 2018).

Psychological accounts foreground engagement, motivation, and self-regulation in learning, with high-attaining learners often understood in terms of differences in engagement and self-concept rather than attainment alone (Subotnik et al., 2011; Zimmerman, 2000). Neuroscientific perspectives emphasise adaptation through appropriately calibrated challenge, consistent with experience-dependent development and plasticity (Howard-Jones, 2014). Educational perspectives focus on how task design structures learning conditions and mediates progression (Biesta, 2010; Ofsted, 2019). Such perspectives suggest that learning is shaped not simply by learner characteristics, but by the relationship between current capability and the cognitive demands of educational tasks. EoC brings these perspectives into dialogue by focusing on the conditions through which challenge is experienced, sustained, and converted into learning and development.

Within this framing, unrealised potential is understood not as an individual deficit but as a structural outcome of persistent misalignment between cognitive readiness and task design. In high-attaining learners, this is typically expressed as insufficient cognitive challenge rather than disengagement (Bjork & Bjork, 2011; Subotnik et al., 2011). EoC responds to common approaches to differentiation that rely on stratified tasks or isolated extension activities through the Low-Threshold High-Ceiling (LTHC) principle. As McClure (2011, p. 1) notes, ‘the content often remains quite simple but the level of thinking required can become very sophisticated’. LTHC operationalises this by embedding access and extension within the same learning task rather than separating them. This reframes differentiation as variation in learning trajectory rather than task allocation, addressing the tendency for HA to be absorbed into generalised models of adaptive teaching (DfE, 2019a; EEF, 2021).

EoC requires a risk of failure and opportunities for success within any task design. Effective challenge requires a balance where tasks are sufficiently demanding to generate cognitive effort, but attainable enough to sustain engagement. This balance is closely linked to motivation and persistence, where overly low or high challenge reduces productive engagement (Subotnik et al., 2011). Where this calibration is absent, challenge becomes either insufficiently demanding or unsustainably difficult, limiting development particularly in HA learners.

Independence in learning is understood in EoC more as a product of structured learning, rather than an assumed learner trait. Independence may involve choices about subject matter, level and approach, as much as working without direct instruction. Where opportunities for calibrated challenge are limited, the development of sustained self-direction is constrained. Independence allows for learners to regulate their thinking, attention, and strategies in response to increasing challenge. This is closely linked to self-regulation, where learners develop control over their learning through progressively demanding tasks. One example is the TASC framework, ‘Thinking Actively in a Social Context’ (Wallace & Adams, 1993). The TASC Wheel conceptualises learning as an iterative cycle in which learners actively plan, monitor, and evaluate problem-solving processes, linking metacognition to progressively structured cognitive challenge and task complexity.

Metacognition provides a key regulatory process linking cognition, engagement, and progression. It refers to learners’ capacity to monitor, evaluate, and adapt their own thinking (Zimmerman, 2000). Despite its importance, ITE provides limited systematic preparation for designing learning environments that actively develop this capacity. This is particularly significant for high-attaining learners, where underachievement is often associated with constrained autonomy rather than task difficulty alone (Subotnik et al., 2011). EoC therefore links cognitive demand with learner regulation, positioning challenge as both a structural and self-regulatory process.

### 3.3. Supporting Conditions for Equality of Challenge

Alongside the core principles of challenge outlined above, EoC incorporates several additional conditions that support access to challenge. These include opportunities to learn alongside age-peers and like-minded peers, the role of novelty and passion in sustaining engagement, and learning beyond the classroom. While less central to the present discussion of ITE, these supporting considerations further illustrate the interdisciplinary nature of EoC and its concern with the wider conditions through which challenge is experienced. Challenge is shaped not only by task design and pedagogy, but also by the wider environments within which learning takes place. As Thomas (2024) notes, ‘the individual child is nested within the context of the school, family, society, and culture’ (p. 251). Challenge is therefore shaped not only by task design and pedagogy, but also by the wider environments within which learning takes place. Educational experiences do not occur in a vacuum, or in isolation, but within wider contexts that can either support or constrain access to challenge.

EoC foregrounds opportunities to learn alongside both age-peers and like-minded peers as contextual conditions for challenge. These ideas are understood across psychology (social comparison and motivation), neuroscience (socially mediated learning and developmental plasticity), and education (peer grouping and subject communities), where interaction with similarly oriented learners can either sustain or constrain engagement with high-level challenge. Within ITE, this suggests that learning is not solely individual, but shaped by access to cognitively aligned peer environments that can either amplify or constrain high-level engagement.

Novelty and passion work as drivers of cognitive-affective engagement. Psychological accounts link novelty to curiosity and intrinsic motivation in learning (Hattie, 2009). Neuroscientific evidence suggests that novelty enhances attentional allocation (Kang et al., 2009), although there are a number of neuromyths around the importance of dopamine and engagement and so caution must be applied (Howard-Jones, 2014). Educational research similarly positions novelty as important for fostering engagement and deep learning within structured pedagogies (Hattie, 2009). Within ITE, this indicates that cognitive challenge depends not only on difficulty, but also on affective engagement, where curiosity and sustained interest help maintain engagement with challenge over time.

Learning beyond the classroom provides a further illustration of the interdisciplinary concerns that underpin EoC. Varied contexts can sustain motivation and build group and self-identity (Winstanley, 2010). Studies show us how hard it can be to link education and neurology, whilst also suggesting that there will be ways to ‘benefit both educators and cognitive neuroscientists, who will gain new perspectives for posing and answering crucial questions about the learning brain’ (Ansari & Coch, 2006, p. 146). Literature on the benefits of learning in different spaces may increasingly be complemented by neuro-educational research, reinforcing its value in helping provide challenge. For many years, using spaces for enrichment, fieldwork, and external learning has been understood as good practice when constitutive of curriculum rather than supplementary to it (Dewey, 1938). Within ITE, this reframes learning opportunities as spatially distributed, where unequal access to enrichment produces systematic variation in cognitive challenge and developmental opportunity.

## 4. Conclusions

The analysis has shown a consistent pattern across policy, disciplinary scholarship, and ITE: GE is not absent, but structurally diminished through conceptual instability, institutional simplification, and fragmented knowledge integration. Across the literature, ‘giftedness’ emerges as a contested construct without stable resolution. This instability is persistent, shaped by contextual dependency and political sensitivity. Ambrose et al. (2012) highlight persistent conceptual incoherence, while Borland (2014) challenges the coherence of categorisation itself. These and other accounts describe a field in which instability is not a temporary problem but an embedded condition.

Within ITE, these tensions are translated into practice through systems that prioritise procedural clarity, standardisation, and accountability. Inspection frameworks and curriculum guidance tend to privilege measurable progression and mid-range attainment, with high-level cognitive demand remaining under-specified. In this context, HA learners are typically absorbed into generic differentiation models rather than explicitly recognised in terms of their cognitive trajectory or developmental need.

Across psychology, neuroscience, and education, relevant knowledge is extensive but insufficiently integrated. Psychological accounts emphasise variation in motivation, engagement, and identity; neuroscientific evidence highlights experience-dependent plasticity and the necessity of calibrated cognitive demand for development; educational frameworks prioritise scalable pedagogical models suited to system-wide implementation. However, these domains remain largely parallel within ITE, limiting the extent to which relevant knowledge informs practice. The consequence is a diluted and uneven conception of educational ‘challenge’. It is often reduced to extension tasks or incremental differentiation rather than understood as a fundamental property of curriculum design and pedagogical intent. Diverse HA learners are therefore neither excluded nor properly addressed, but positioned within broad categories that can obscure meaningful learning. Unequal access to enrichment produces systematic variation in cognitive challenge and developmental opportunity; this is not simply a matter of provision, but of educational justice.

The contribution of this paper is therefore not to resolve longstanding debates surrounding giftedness, but to offer a way of thinking about challenge, learner variability, and educational entitlement that remains meaningful despite them. It is within this context that Equality of Challenge is proposed as a way of reframing the issue in terms of entitlement to appropriate challenge across the full range of learners. EoC is not simply a conceptual addition to existing ITE frameworks. It is a moral reorientation. It asserts that all learners are entitled not only to access education, but to encounter education that is appropriately demanding, intellectually substantive, and developmentally meaningful. Lack of challenge may result in technical inefficiency. More fundamentally, however, it represents a failure of educational justice.

From this perspective, ITE cannot remain neutral on the question of challenge. If teachers’ identities continue to be formed with existing priorities, we risk reproducing a system in which some learners are consistently presented with work that is insufficiently challenging. This is not a marginal issue of provision, but a structural question about what counts as a worthwhile educational experience.

The implication is clear. Without a coherent framework for understanding and enacting cognitive variability, teacher education cannot reliably deliver on its ethical responsibility to ensure meaningful learning across the full range of learners. The central proposition of EoC is that challenge should be understood as an educational entitlement rather than a reward for prior attainment. Equality of Challenge therefore offers a way of articulating that responsibility through a commitment to every learner’s entitlement to appropriately demanding educational experiences.

## Data Availability

No new data were created in this study. Data sharing is therefore not applicable to this article.

## Abbreviations

The following abbreviations are used in this manuscript:

DfE	Department for Education (England, UK)
DME	Dual or Multiple Exceptionalities (also known as ‘Twice Exceptional’)
EEF	Education Endowment Foundation (UK)
EoC	Equality of Challenge
GE	Gifted Education
HA	High Ability
ITE	Initial Teacher Education (also known as ‘ITT—Initial Teacher Training’)
LTHC	Low-Threshold High-Ceiling
NACE	National Association for Able Children in Education (UK)
OECD	Organisation for Economic Co-operation and Development
SEN/D	Special Educational Needs/Disability

## References

[B1-behavsci-16-01334] Ambrose D., Sternberg R. J., Sriraman B. (2012). Confronting dogmatism in gifted education.

[B2-behavsci-16-01334] Ansari D., Coch D. (2006). Bridges over troubled waters: Education and cognitive neuroscience. Trends in Cognitive Sciences.

[B3-behavsci-16-01334] Ball S. J. (2003). The teacher’s soul and the terrors of performativity. Journal of Education Policy.

[B4-behavsci-16-01334] Biesta G. J. J. (2010). Good education in an age of measurement: Ethics, politics, democracy.

[B5-behavsci-16-01334] Bjork E. L., Bjork R. A., Gernsbacher M. A., Pew R. W., Hough L. M., Pomerantz J. R. (2011). Making things hard on yourself, but in a good way: Creating desirable difficulties to enhance learning. Psychology and the real world: Essays illustrating fundamental contributions to society.

[B6-behavsci-16-01334] Borland J. H., Sternberg R. J., Davidson J. E. (2005). Gifted education without gifted children. Conceptions of giftedness.

[B7-behavsci-16-01334] Borland J. H., Plucker J. A., Callahan C. M. (2014). Identification of gifted students. Critical issues and practices in gifted education: What the research says.

[B8-behavsci-16-01334] Dai D. Y., Pfeiffer S. I., Shaunessy-Dedrick E., Foley-Nicpon M. (2018). A history of giftedness: A century of quest for identity. APA handbook of giftedness and talent.

[B9-behavsci-16-01334] Department for Education (2011). Teachers’ standards.

[B10-behavsci-16-01334] Department for Education (2015). Carter review of initial teacher training (ITT).

[B11-behavsci-16-01334] Department for Education (2019a). Early career framework.

[B12-behavsci-16-01334] Department for Education (2019b). Initial Teacher Training (ITT) core content framework.

[B13-behavsci-16-01334] Department for Education (2021). Teachers’ standards: Guidance for school leaders, school staff and governing bodies.

[B14-behavsci-16-01334] Department for Education (2024). 2024 updated combined framework: Initial teacher training and early career framework (ITTECF).

[B15-behavsci-16-01334] Dewey J. (1938). Experience and education.

[B16-behavsci-16-01334] Education Endowment Foundation (2018). New guidance report published: How to develop pupils’ metacognitive skills.

[B17-behavsci-16-01334] Education Endowment Foundation (2021). ECF—Exploring the evidence: ‘Adaptive teaching’ and effective diagnostic assessment.

[B18-behavsci-16-01334] Freeman J. (2010). Gifted lives: What happens when gifted children grow up.

[B19-behavsci-16-01334] Glazzard J., Stones S. (2020). Mythbusting for trainee teachers.

[B20-behavsci-16-01334] Gross M. U. M. (2004). Exceptionally gifted children.

[B21-behavsci-16-01334] Hattie J. (2009). Visible learning: A synthesis of over 800 meta-analyses relating to achievement.

[B22-behavsci-16-01334] Howard-Jones P. A. (2014). Neuroscience and education: Myths and messages. Nature Reviews Neuroscience.

[B23-behavsci-16-01334] Immordino-Yang M. H., Darling-Hammond L., Krone C. (2019). Nurturing nature: How brain development is inherently social and emotional, and what this means for education *(EdWorkingPaper: 19-106)*.

[B24-behavsci-16-01334] Kang M. J., Hsu M., Krajbich I. M., Loewenstein G., McClure S. M., Wang J. T., Camerer C. F. (2009). The wick in the candle of learning: Epistemic curiosity activates reward circuitry and enhances memory. Psychological Science.

[B25-behavsci-16-01334] Loft P., Danechi S. (2020). Support for more able and talented children in schools (UK) *(House of Commons Library Briefing Paper No. 9065)*.

[B26-behavsci-16-01334] Lowe H. (2017). Guidance for parents and carers: Supporting your child with high ability.

[B27-behavsci-16-01334] McClure R. (2011). Low threshold high ceiling tasks.

[B28-behavsci-16-01334] Melander A. (2025). Distributive values and disadvantaged potential high achievers, in ‘Educational Decision-Making: Combining Evidence and Values’ (Suite of Papers). Journal of Philosophy of Education.

[B29-behavsci-16-01334] Merry M. S. (2008). Educational justice and the gifted. Theory and Research in Education.

[B30-behavsci-16-01334] Montacute R. (2018). Potential for success: Fulfilling the promise of highly able students in secondary schools.

[B31-behavsci-16-01334] OECD (2019). TALIS 2018 results (volume I): Teachers and school leaders as lifelong learners.

[B32-behavsci-16-01334] Ofsted (2015). School inspection handbook.

[B33-behavsci-16-01334] Ofsted (2019). Education inspection framework: Overview of research.

[B34-behavsci-16-01334] Orchard J., Kelly L., Winstanley C. (2020). ‘Head’ and ‘heart’ work. Studia Paedagogica.

[B35-behavsci-16-01334] Pfeiffer S. I. (2013). Serving the gifted: Evidence-based clinical and psychoeducational practice.

[B36-behavsci-16-01334] Sternberg R. J., Sternberg R. J., Davidson J. E. (2005). The WICS model of giftedness. Conceptions of giftedness.

[B37-behavsci-16-01334] Sternberg R. J. (2023). Giftedness does not reside within a person: Defining giftedness in society is a three-step process. Roeper Review.

[B38-behavsci-16-01334] Subotnik R. F., Olszewski-Kubilius P., Worrell F. C. (2011). Rethinking giftedness and gifted education: A proposed direction forward based on psychological science. Psychological Science in the Public Interest.

[B39-behavsci-16-01334] Thomas M. S. C. (2024). What do teachers need to know about neuroscience?. MedUNAB [Internet].

[B40-behavsci-16-01334] Thomas M. S. C., Ansari D., Knowland V. C. P. (2019). Annual Research Review: Educational neuroscience: Progress and prospects. Journal of Child Psychology and Psychiatry.

[B41-behavsci-16-01334] Thomas M. S. C., Arslan Y. (2025). Why does the brain matter for education?. British Journal of Educational Psychology.

[B42-behavsci-16-01334] Thomas M. S. C., Howard-Jones P., Dudman-Jones J., Palmer L. R. J., Bowen A. E. J., Perry R. C. (2024). Evidence, policy, education and neuroscience—The state of play in the UK. Mind, Brain, and Education.

[B43-behavsci-16-01334] Wallace B., Adams H. (1993). TASC: Thinking actively in a social context.

[B44-behavsci-16-01334] Wiliam D. (2018). Embedded formative assessment.

[B45-behavsci-16-01334] Winstanley C. (2004). Too clever by half: A fair deal for gifted children.

[B46-behavsci-16-01334] Winstanley C., Hand M., Winstanley C. (2008). Philosophy and the development of critical thinking. Philosophy in schools.

[B47-behavsci-16-01334] Winstanley C. (2010). The ingredients of challenge.

[B48-behavsci-16-01334] Winstanley C. (2018). Deep thinking and high ceilings: Using philosophy to challenge ‘more able’ pupils. Journal of Philosophy in Schools.

[B49-behavsci-16-01334] Winstanley C. (2019). Breaking barriers: Supporting more able learners.

[B50-behavsci-16-01334] Worrell F. C., Subotnik R. F., Olszewski-Kubilius P., Dixson D. D. (2019). Gifted students. Annual Review of Psychology.

[B51-behavsci-16-01334] Zimmerman B. J., Boekaerts M., Pintrich P., Zeidner M. (2000). Attaining self-regulation: A social cognitive perspective. Handbook of self-regulation.

